# The Roles of Tumor-Derived Exosomes in Cancer Pathogenesis

**DOI:** 10.1155/2011/842849

**Published:** 2011-11-30

**Authors:** Chenjie Yang, Paul D. Robbins

**Affiliations:** Department of Microbiology and Molecular Genetics, University of Pittsburgh School of Medicine, Pittsburgh, PA 15219, USA

## Abstract

Exosomes are endosome-derived, 30–100 nm small membrane vesicles released by most cell types including tumor cells. They are enriched in a selective repertoire of proteins and nucleic acids from parental cells and are thought to be actively involved in conferring intercellular signals. Tumor-derived exosomes have been viewed as a source of tumor antigens that can be used to induce antitumor immune responses. However, tumor-derived exosomes also have been found to possess immunosuppressive properties and are able to facilitate tumor growth, metastasis, and the development of drug resistance. These different effects of tumor-derived exosomes contribute to the pathogenesis of cancer. This review will discuss the roles of tumor-derived exosomes in cancer pathogenesis, therapy, and diagnostics.

## 1. Introduction

Membranous vesicle shedding from live cells was first observed in the early 1980s and was proposed to be a mechanism through which cells discard inert debris [1–4]. Different types of membrane vesicles are secreted by cells, formed either at the surface of a blebbing plasma membrane or inside internal cellular compartments [5]. Among them, a population of nanosized membrane vesicles, termed “exosomes,” has gained interest for their pleiotropic biological activity. Exosomes are defined as vesicles formed by “inward/reverse budding” of the limiting membrane of the multivesicular bodies (MVBs) in the late endocytic compartment and released upon the fusion of MVB with the plasma membrane [6, 7]. They are characterized by a size of 30–100 nm in diameter and a density of 1.13–1.19 g/mL in a sucrose gradient and can be sedimented at 100,000 ×g [5, 8]. Exosomes typically show a “cup-shaped” or “saucer-like” morphology when analyzed by electron microscopy. Exosome secretion is observed from most cell types under both physiological and pathological conditions, especially tumor cells and hematopoietic cells including reticulocytes [2, 4, 9, 10], dendritic cells (DCs) [11], B and T lymphocytes [12–15], platelets [16], mast cells [17, 18], and macrophages [19]. In addition, exosomes are also released by epithelial cells [20], fibroblasts [21], astrocytes, and neurons [22]. The extent of exosome secretion can be modulated in different cell types by either ligand cognition or stress conditions. For example, radiation treatment is able to increase the level of exosome secretion by tumor cells, a process possibly involving the activation of p53 and the subsequent upregulation of the transmembrane protein tumor suppressor-activated pathway 6 (TsAP6) [21, 23].

Exosomes contain cytosolic and membrane proteins derived from the parental cells. The protein content largely depends on their cellular origin and are generally enriched for certain molecules, including targeting/adhesion molecules (e.g., tetraspanins, lactadherin and intergrins), membrane trafficking molecules (e.g., annexins and Rab proteins), cytoskeleton molecules (e.g., actin and tubulin), proteins involved in MVB formation (e.g., Alix, Tsg101 and clathrin), chaperones (e.g., Hsp70 and Hsp90), signal transduction proteins (e.g., protein kinases, 14-3-3, and heterotrimeric G proteins) and cytoplasmic enzymes (e.g., GAPDH, peroxidases, and pyruvate kinases) [5, 8, 24]. Antigen presenting cell- (APC-) derived exosomes are also enriched in antigen-presenting molecules including MHC class I and class II complexes and costimulatory molecules [25]. Tumor-derived exosomes usually contain tumor antigens as well as certain immunosuppressive proteins such as FasL, TRAIL, or TGF-*β* [26]. In addition to proteins, functional RNA molecules including mRNA and microRNAs have also been identified in exosomes [27–29]. 

Exosomes are now recognized as important mediators of cell-to-cell communication [30]. However, how these vesicles interact with and regulate the function of target cells remains largely unknown. Several types of interactions are proposed based on indirect evidence and *in vitro* studies, including (1) binding of vesicles to the surface of a recipient cell through exosomal adhesion molecules, or phosphatidylserine (PS)/lysophosphatidylcholine and cellular receptors (e.g., LFA1, TIM1 and TIM4); (2) direct fusion of vesicles with recipient plasma membrane after adhesion; or (3) internalization of vesicles into endocytic compartments through receptor-mediated endocytosis or phagocytosis [5]. Also, the symmetrical phatidylethanolamine repartitions in exosome membranes may facilitate their absorption, but not fusion with target cells such as DCs [31]. 

The interaction between exosomes and target cells can lead to direct stimulation of target cells via surface-expressed growth factors or bioactive lipids, transfer of membrane receptors, or delivery of proteins to target cells. Also, the presence of mRNA and microRNA, termed “exosomal shuttle RNA,” in exosomes suggests that genetic material exchange could be an additional level of exosome-mediated communication between cells [27].

There is still some confusion in describing different types of vesicles secreted by cells. The terms “exosomes,” “microvesicles,” and “membrane particles” are sometimes used interchangeably. Generally, the term “microvesicles” refers to vesicles shed from the plasma membrane, have a relatively larger size (100–1000 nm) than exosomes and can be sedimented at 10,000 ×g. The term “membrane particles” refers to vesicles that also originate from plasma membrane, but have a small size similar to exosomes [5]. In this review, we will focus specifically on the various effects of exosomes on tumorigenesis.

## 2. Antitumorigenic Role of Tumor-Derived Exosomes

### 2.1. Immunogenic Properties and Tumor Exosome-Based Cancer Vaccines

The protein composition of exosomes largely reflects that of their parental cells and thus shows cell-type specificity. In particular, tumor-derived exosomes contain tumor-specific antigens expressed in the parental tumor cells. Enrichment of tumor antigens such as melan-A [32], Silv [33], carcinoembryonic antigen (CEA) [34], and mesothelin [35] is observed in tumor-derived exosomes when compared with whole cell lysates [26]. The observation that most tumor cells release exosomes containing tumor antigens suggests that tumor exosome-based cancer vaccines could be developed. Indeed, tumor-derived exosomes have been used as a source of tumor antigens to pulse DCs, resulting in the transfer of tumor antigens to DCs that were able to induce CD8+ T cell-dependent antitumor effects in mice [33]. In a similar human *ex vivo* model system, DCs pulsed with exosomes derived from malignant effusions expressing tumor antigens cross-present the antigens to antigen-specific cytotoxic T lymphocytes (CTLs) [32]. Recently, it was reported that tumor exosome-loaded DCs effectively elicited tumor-specific CD8+ CTL response against autologous tumor cells in patients with malignant gliomas [36]. 

Direct application of tumor-derived exosomes for the enhancement of antitumor immunity also has been investigated. It was reported that tumor-derived exosomes could induce specific antitumor responses when the parental tumor cells were genetically modified to express pro-inflammatory cytokines such as IL-18, IL-12, and IL-2 [37–39] or when the parental tumor cells were subjected to stress conditions. For example, heat-shocked lymphoma cells release exosomes with increased levels of MHC and co-stimulatory molecules and induce efficient antitumor T cell immunity [40]. Additionally, exosomes derived from heat-shocked tumor cells were observed to contain elevated levels of Hsp70 and elicit Th1-polarized immune responses *in vitro* and *in vivo* in both autologous and allogeneic murine models, suggesting that these exosomes can stimulate antitumor immunity in an MHC-independent manner [41]. Moreover, it was reported that exosomes derived from tumor cells engineered to express membrane-bound Hsp70 stimulate Th1 and CTL antitumor immunity more efficiently than those derived from heat-shocked tumor cells [42]. Heat-stressed tumor cells were also found to release exosomes with enriched chemokines that could attract and activate DCs and T cells more potently and induce specific antitumor immune response more efficiently than exosomes from untreated tumor cells [43]. Furthermore, surface targeting of antigens to exosome membranes can enhance the immunogenicity of tumor-derived exosomes, as membrane targeting of the superantigen staphylococcal enterotoxin A (SEA) [44] or chicken egg ovalbumin (OVA) [45] resulted in enhanced CTL activity and delayed tumor growth.

The promising results obtained in animal tumor models led to several phase I clinical trials using tumor-derived exosomes or exosome-pulsed DCs as cancer vaccines [46, 47]. However, it is important to note that in animal experiments, effective antitumor immune responses were mostly achieved when tumor-derived exosomes were loaded onto matured APCs or were modified to contain high levels of pro-inflammatory factors or stress proteins. The representative studies on the immunogenicity of tumor-derived exosomes and tumor exosome-based cancer vaccines are listed in Table 1. 

### 2.2. Induction of Tumor Cell Apoptosis

In addition to the potential immunostimularoty effects, a proapoptotic function of tumor-derived exosomes directly on tumor cells was also reported. Exosome-like vesicles produced by human pancreatic tumor cells were reported to increase Bax and decrease Bcl-2 expression, inducing tumor cells toward mitochondria apoptotic pathway. These exosomes also induced phosphatase and tensin homolog (PTEN) and glycogen synthase kinase-3*β* (GSK-3*β*) activation and decreased pyruvate dehydrogenase activity in treated cells, sequestered *β*-catenin-dependent survival pathway, and counteracted the constitutively activated phosphatidylinositol 3-kinase/Akt survival pathway to drive tumor cells toward apoptosis [48]. The interaction of these exosomes with pancreatic cancer cells also led to decreased expression of the intranuclear target of the Notch-1 signaling pathway, thereby inhibiting the Notch-1 survival pathway and activating the apoptotic pathway [49]. 

Despite the potential antitumor effects of tumor-derived exosomes, it is still unclear whether the constant production of exosomes by tumor cells is beneficial or harmful for their own survival *in vivo*. Notably, in cancer patients with advanced disease, tumor-derived exosomes are produced abundantly in the tumor microenvironment, however effective immunostimulatory or antitumor effects of these vesicles are rarely observed. In fact, there is substantial evidence supporting a role of tumor-derived exosomes in preventing antitumor immune responses and promoting tumorigenesis.

## 3. Protumorigenic Role of Tumor-Derived Exosomes

### 3.1. Immunosuppressive Properties

The observation that membrane vesicles shed from murine melanoma cell lines inhibited the expression of the immune response region-associated antigen by macrophages provided early evidence that tumor-derived membrane vesicles is a possible mechanism whereby tumor-bearing hosts become immunocompromised [50]. More recently, diverse immunosuppressive effects of tumor-derived exosomes have been identified. Tumor-derived exosomes were shown to directly suppress the activity of effector T cells. Certain tumor cell lines can produce exosomes expressing death ligand such as FasL and TRAIL, both of which can trigger the apoptotic death of activated T cells [51, 52]. Additionally, Epstein-Barr Virus- (EBV-) infected nasopharyngeal carcinoma (NPC) was shown to release exosomes containing high amounts of galectin-9, which induces apoptosis of mature Th1 lymphocytes when interacting with the membrane receptor Tim-3. These exosomes prevent galectin-9 from being proteolytically cleaved and thus induce massive apoptosis of EBV-specific CD4+ cells [53]. Moreover, ovarian tumor-derived exosomes were found to down-modulate CD3-*ζ* chain expression and impair TCR signaling [54], suggesting that tumor-derived exosomes can also downregulate T cell function in addition to direct killing. In addition, NKG2D-dependent cytotoxicity of NK cells and CD8+ T cells was inhibited by NKG2D ligand-containing exosomes derived from human breast cancer and mesothelioma cell lines [55, 56]. Similarly, murine mammary carcinoma exosomes were shown to promote tumor growth *in vivo* by suppressing NK cell function [57]. Taken together, these observations suggest that tumor-derived exosomes can negatively regulate the effector arm of the immune system, in particular T cells and NK cells. 

Tumor-derived exosomes can also target myeloid cells to modulate their differentiation and function. Exosomes derived from human melanoma cell lines and colorectal carcinoma cell lines were shown to skew monocyte differentiation into DCs toward the generation of myeloid-derived suppressor cells (MDSCs) and exert TGF-*β*1 mediated suppressive activity on T cells *in vitro*. Interestingly, significant expansion of MDSC-like CD14+HLA-DR-/low and TGF-*β*-secreting cells was also found in the peripheral blood of late-stage melanoma patients and high levels of MDSCs is usually associated with poor responses to tumor vaccines [58]. Similar effects were observed in mouse models where exosomes produced by murine mammary carcinoma cells and melanoma cells targeted CD11b+ myeloid precursors in the bone marrow (BM) and inhibited the differentiation of BMDCs by inducing IL-6 in these precursor cells [59]. These myeloid cells were found to switch their differentiation pathway toward an MDSC phenotype and promote tumor growth, dependent on the prostaglandin E2 and TGF-*β* molecules present on tumor-derived exosomes [60]. MyD88 also appears to play a pivotal role in melanoma exosome-mediated MDSC expansion and tumor metastasis [61]. Similarly, the membrane-associated Hsp72 on tumor-derived exosomes was reported to mediate STAT3-dependent immunosuppressive function of MDSCs by triggering STAT3 activation in a Toll-like receptor- (TLR-) 2/MyD88-dependent manner [62], although the role of TLR2 in this process remains controversial [63, 64].

The effect of tumor-derived exosome on BM cells is thought to be a coevolutionary strategy of the primary tumor and the tumor microenvironment [65]. Alteration of BM cell behavior by tumor-derived exosomes can be mediated by proteins or by transfer of genetic materials, such as mRNA and microRNA, between tumor cells and BM cells, thereby influencing the function of future populations of BM cells. RNA transfer to BM cells by microvesicles released from other tissue/cell sources and the transcription of tissue-specific mRNA in BM cells has been observed [66, 67], suggesting that a similar effect also can be mediated by tumor-derived exosomes. 

In addition, tumor-derived exosomes can also support the function of regulatory T (Treg) cells. For example, human tumor-derived exosomes were found to selectively impair the IL-2 response to cytotoxic effector cells while supporting Treg cell activities through a TGF-*β*-dependent mechanism [35]. Tumor-derived exosomes were also reported to induce, expand, and upregulate the suppressor functions of human Treg cells as well as enhance their resistance to apoptosis via a TGF-*β*- and IL-10-dependent mechanism [68]. A similar effect was observed with exosomes derived from the malignant effusion of cancer patients as these exosomes, most of which have a tumor origin, helped maintain the number and suppressive function of Treg cells [69].

Given that tumor-derived exosomes are capable of altering APC function and enhancing regulatory cell activity while at the same time are a source of tumor antigen, it is tempting to speculate that tumor-derived exosomes may also have the ability to promote tolerance to tumor-specific antigens. Indeed, we have demonstrated that tumor-derived exosomes bearing a model tumor antigen were able to induce antigen-specific immunosuppression in a murine delayed-type hypersensitivity model. We proposed a mechanism that tumor-derived exosomes provide tumor antigens to DCs as well as condition DCs toward a suppressive/tolerogenic phenotype, resulting in the downregulation of antigen-specific immune responses [70].

### 3.2. Facilitation of Tumor Invasion and Metastasis

In addition to attenuating different branches of the antitumor immunity to help tumor cells survive immunosurvelliance, tumor-derived exosomes have also been implicated in facilitating tumor invasion and metastasis. By stimulating angiogenesis, modulating stromal cells, and remodeling extracellular matrix, tumor-derived exosomes have been found to contribute to the establishment of a premetastatic niche, generating a suitable microenvironment in distant metastatic sites [65]. 

Early proteomic analysis of mesothelioma cell-derived exosomes detected the presence of strong angiogenic factors that can increase vascular development in the neighborhood of tumor [71]. Melanoma-derived exosomes were also found to stimulate endothelial signaling important for tissue matrices remodeling and endothelial angiogenesis [72]. Moreover, it was recently reported that melanoma exosomes injected locally preferentially homed to sentinel lymph nodes and prepared the lymph nodes to become remote niches conducive to the migration and growth of melanoma cells through the induction of molecular signals for melanoma cell recruitment, extracellular matrix deposition, and vascular proliferation [73]. Consistent with these observations, it was reported that mice pretreated with melanoma exosomes have a significant acceleration of melanoma metastasis in the lung [61].

Tetraspanins, which are constitutively enriched in exosomes, have been found to contribute to exosome-mediated angiogenesis. It was reported that exosomes derived from a pancreatic tumor line overexpressing D6.1A, a tetraspanin associated with poor prognosis in patients with gastriointestinal cancer, strongly induced endothelial cell branching *in vitro* and angiogenesis *in vivo* in a rat model [74]. Tumor-derived D6.1A stimulates the secretion of matrix metalloproteinase and urokinase-type plasminogen activator, enhances the expression of vascular endothelial growth factor expression in fibroblasts, and upregulates the expression of endothelial growth factor receptor as well as D6.1A in sprouting endothelium. Moreover, the D6.1A-expressing cell promoted angiogenesis independent of cell-cell contact, highlighting the potential role of D6.1A-enriched tumor-derived exosomes in inducing systemic angiogenesis. Recently, exosomal Tspan8 (D6.1A) was found to contribute to the selective recruitment of proteins and mRNA into exosomes, including CD106 and CD49d, both of which were implicated in the binding and internalization of exosomes by endothelial cells. Induction of several angiogenesis-related genes, together with enhanced endothelial cell proliferation, migration sprouting and maturation of endothelial cell progenitors, were seen upon exosome internalization [75]. Tumor-derived exosomes were also found to incorporate the Notch ligand Delta-like 4 (Dll4) and transfer the Dll4 protein into the cell membrane of host endothelial cells, resulting in the inhibition of Notch signaling and the switch of endothelial cell phenotype toward tip cells. This further results in an increase in vessel density *in vitro* and an increase in branching* in vivo * [76].

Another pronounced effect of tumor-derived exosomes is their ability to modulate the function of stromal cells such as fibroblasts. It was recently shown that exosomes produced by a certain type of cancer cells contain TGF-*β* on their surface in association with betaglycan and can trigger SMAD-dependent signaling. Exosomal delivery of TGF-*β* is capable of driving the differentiation of fibroblasts into myofibroblasts, whose enrichment in solid tumor represents an altered stroma that usually supports tumor growth, vascularization, and metastasis. Exosomal TGF-*β* delivery is also qualitatively different from soluble TGF-*β* in that they induce a more significant elevation of fibroblast FGF2 production [77]. These observations suggest another protumorigenic role of tumor-exosomal TGF-*β* in addition to their immunosuppressive functions. However, it was also noted that TGF-*β* is not universally present on exosomes derived from all cancer cells. 

Furthermore, exosomes shed by gynecologic neoplasias, including ovarian cancer and breast cancer cells, were found to contain metalloproteinases that have proteolytic activity. These exosomes can increase extracellular matrix degradation and augment tumor invasion into the stroma [78–80]. It was suggested that CD44 is required for the assembly of a soluble matrix which may serve as an exosome carrier and/or a reservoir for growth factors, chemokines, and proteases needed for tumor cell embedding and growth. Selective knockdown of CD44 resulted in a striking reduction of the metastasizing capacity of the highly metastatic tumor in a rat pancreatic adenocarcinoma model [81]. 

Interestingly, tumor-derived microvesicles, which are mostly shed from tumor plasma membrane, were found to have certain effects similar to exosomes, such as stimulating angiogenesis [82, 83], modifying stromal cells [84], and degrading extracellular matrix [85–87], possibly because that they have comparable compositions and that the proteins involved are present on both types of vesicles. However, the vesicles reported to have a procoagulant effect that correlates with an increased risk of cancer-associated thromboembolism have been mostly microvesicles, rather than exosomes, likely because the tissue factors and other contents with procoagulant activity such as PS and Mucin 1 mostly reside in the cell surface membrane. Those microvesicles are also thought to play an important role in supporting tumor growth by inducing the local fibrin deposits associated with many solid tumors [88–92]. 

### 3.3. Transport of RNAs and Proteins for Tumor Survival and Growth

The intercellular exchange of proteins and genetic materials via exosomes is a potentially effective approach for cell-to-cell communication within the tumor microenvironment [93]. In particular, transport of mRNAs and microRNAs, from tumor cells to neighboring cells could have significant effects on tumorigenesis. Glioblastoma-derived exosomes were reported to transport mRNA into recipient cells where it is functionally translated. These exosomes stimulated glioma cell proliferation and promoted tumor growth [28]. The let-7 microRNA family was found to be selectively released in exosomes in a metastatic gastric cancer cell line. Since the *let-7* genes target oncogenes including RAS and HMGA2 and generally play a tumor-suppressor role, the release of let-7 microRNA via exosomes could deliver oncogenic signals and promote metastasis [94]. Moreover, exosomes can also be utilized by human tumor virus for disseminating viral materials. For example, exosomes released from NPC cells with latent EBV infection contain EBV latent membrane protein 1 (LMP1) and viral microRNAs. These exosomes were able to transfer LMP1 into recipient cells and activate growth-signaling pathway [95]. Similarly, it was reported that the viral BART miRNAs are released from EBV-infected NPC cells into exosomes. These viral microRNAs could be detected in blood plasma samples from NPC xenografted nude mice as well as NPC patients, suggesting that exosomes enable these viral miRNAs to diffuse from the tumor site to the peripheral blood [96].

Tumor-derived exosomes may also transport apoptosis-inhibitory proteins induced under stress conditions to promote tumor survival. For example, survivin, a member of the inhibitor of apoptosis protein family, can be absorbed by cancer cells from extracellular media and inhibit their apoptosis following genotoxic stress as well as increase their replicative and metastatic ability [97]. It was found that survivin was released into exosomes from cervical carcinoma cells at a significantly higher level after irradiation, suggesting a potential exosome-mediated self-protective mechanism of these cancer cells [98].

### 3.4. Drug Interference

The protumorigenic role of tumor-derived exosomes is also reflected by their active participation in drug resistance through several mechanisms. One mechanism is by drug exportation via the exosome pathway. In human ovarian carcinoma cells that stably acquired resistance to the cancer chemotherapy drug cisplatin, the lysosome compartment, where the drug usually accumulates, was reduced with more exosomes released compared to cisplatin-sensitive cells. Moreover, when the cells were loaded with cisplatin, exosomes released from cisplatin-resistant cells contained 2.6-fold more platinum than those released from cisplatin-sensitive cells, suggesting that exosome secretion can be utilized by cancer cell to export anticancer drugs [99]. A similar effect was also observed in melanosomes, a type of lysosome-related organelles in pigmented cells such as melanoma cells [100]. One of the mechanisms by which lysosomal vesicles sequester cytotoxic drugs is increased acidification and treatment with proton pump inhibitors inhibited the acidification process and increased the sensitivity of tumor cells to chemotherapy drugs [101]. In addition, exosomes can also function to neutralize antibody-based drugs. Exosomes secreted by HER2-overexpressing breast carcinoma cell lines express a full-length HER2 molecule, enabling them to bind to the HER2 antibody Trastuzumab both *in vitro* and *in vivo*. The exosome-antibody interactions inhibit the overall effect of Trastuzumab on the proliferation of cancer cells by reducing antibody binding to cancer cells [102]. Such antibody sequestration was also demonstrated to reduce the antibody-dependent cytotoxicity effect on tumor cells by immune effector cells [103].

**Figure 1:**
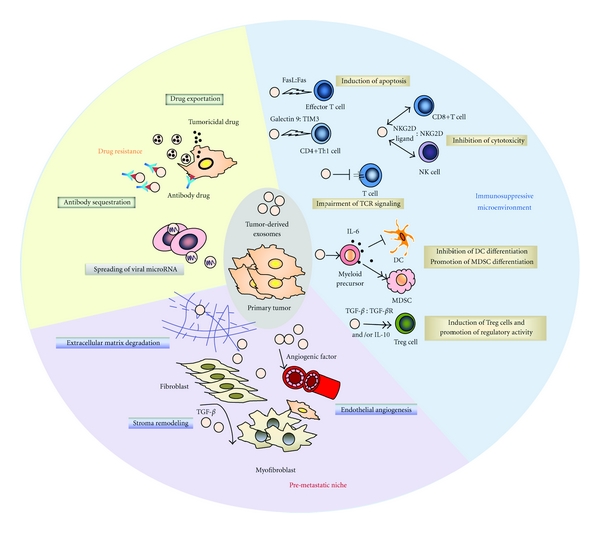
The protumorigenic role of tumor-derived exosomes. Tumor-derived exosomes help create an immunosuppressive tumor microenvironment by inducing apoptosis and impairing the function of effector T cells and NK cells, skewing DC differentiation into MDSCs as well as promoting Treg cell activity. They also contribute to the establishment of a pre-metastatic niche by enhancing angiogenesis, remodeling stromal cells, and promoting extracellular matrix degradation. Tumor-derived exosomes also function as delivery vehicles to transfer microRNA and mRNA to neighboring cells. Moreover, tumor-derived exosomes can help tumor cells develop drug resistance by exporting tumoricidal drugs or neutralizing antibody-based drugs.

Taken together, tumor-derived exosomes exert pro-tumorigenic effects via pleiotropic mechanisms (Figure 1). However, it is important to note that each of the numerous effects of exosomes reported was observed from exosomes derived from only a few of a wide variety of cancerous cell lines or types. Whether exosomes derived from a given tumor will have the sufficient complexity to confer multiple suppressive functions still needs to be determined [104]. It is likely that the predominant regulatory role of exosomes depends on their molecular phenotype and cell specificity. In addition, environmental factors could also play an important role in determining the behavior and immunological impact of tumor-derived exosomes.

## 4. Clinical Relevance of Tumor-Derived Exosomes

As discussed above, tumor-derived exosome-pulsed DCs, tumor-derived exosomes, and exosomes isolated from malignant ascites all have been investigated for their ability to elicit antitumor immune response in patients. However, although these clinical approaches appear to be safe, there has been a lack of clinical efficacy of exosome-based vaccines in contrast to the promising results obtained in many animal tumor models. Because of their potential immunosuppressive properties, direct administration of tumor-derived exosomes may actually result in promoted tumor growth. Therefore, clinical studies have focused on the use of tumor-derived exosome-loaded mature DCs [46] or ascites-derived exosomes [47], which may include both APC- and tumor-derived exosomes, together with proinflammatory factors. Still, the limited number of clinical trials and patients recruited prevents a conclusive evaluation of their efficacy and prospect.

The protumorigenic potential of tumor-derived exosomes in cancer patients is supported by the observations that in patients with breast or ovarian cancer, the level of circulating exosomes and exosomes with tumor markers is much higher than nonmalignant individuals and increases with tumor progression [29, 105], and that exosomes isolated from the sera of patients with oral or ovarian cancer can impair T lymphocytes function and induce their apoptosis [54, 106]. Therefore, it has been proposed that removing immunosuppressive tumor-derived exosomes from the blood circulation of a cancer patient would improve antitumor immune response and delay the progression and spread of malignancy. A novel hollow-fiber cartridge (Hemopurifier) system which is able to selectively deplete circulating virus using a lectin-based resin with high affinity for glycosylated viral surface proteins was developed by the San Diego biotechnology company Aethlon Medical [107]. Effective removal of HIV particles has been demonstrated [108–110] and this system has become an attractive device for depletion of exosomes, which have a size similar to viral particles and are also highly glycosylated on their membrane proteins. The selective removal of exosomes can be enhanced by attaching antibodies against exosome surface proteins onto the resin of the cartridge. However, there are still technical barriers in how to carefully distinguish tumor-derived from nontumor-derived exosomes and concerns such as the physiological outcome of removing all exosome-like vesicles in the blood. 

On the other hand, tumor-derived exosomes containing tumor-specific protein and microRNA profiles have been proposed to be cancer diagnostic markers. Early detection of cancer could be easily performed using exosomes isolated from body fluids such as blood plasma, serum, and urine. Evidence supporting this approach include: (1) ovarian cancer-associated expression of claudin proteins can be detected in the circulating vesicles of a majority of ovarian cancer patients [111], (2) in breast cancer patients increasing levels of circulating vesicles expressing CEA and the cancer antigen 15-3 is correlated with increasing size of tumors [105], (3) exosomes expressing tumor markers can be isolated from the sera of ovarian cancer patients and the amount increases along with tumor progression [29]; and (4) in glioblastoma patients, mRNA variants and microRNAs characteristic of gliomas could be detected in serum vesicles [28]. However, it was also found that not in all cases tumor-derived exosomes were present in the blood circulation [112]. In a study on tumor-derived exosomes in the serum of glioblastoma patients, tumor-specific EGFRvIII was detected in serum exosomes in 7 out of 25 patients [28]. We recently demonstrated that tumor-derived exosomes with a chimeric membrane surface tag could not be detected in plasma-derived exosomes of mice bearing subcutaneous melanoma, possibly due to the rapid uptake of tumor-derived exosomes by APCs in the tumor microenvironment before they have access to the blood circulation (unpublished data). Therefore, different types of tumor and possibly different tumor growth patterns may both affect the accumulation of tumor-derived exosomes in peripheral circulation. Thus cautious interpretation is needed when using the presence of tumor-derived exosomes in body fluids as cancer diagnostic markers.

## 5. Conclusion

Increasing evidence suggests that tumor-derived exosomes can confer either antitumorigenic or protumorigenic effects. These seemingly controversial effects can be the results of complex interactions between exosomes, responding cells, and environmental factors. In cancer patients, the immunostimulatory or immuosuppressive effects of tumor-derived exosomes may also depend on the stage of cancer progression as well as the immune status. Notably, as close replicas of their parental cells, tumor-derived exosomes are well positioned to transmit the detrimental effects of tumor cells onto the immune system to facilitate their survival, growth, and metastasis. Therefore, a better understanding of the roles of tumor-derived exosomes in cancer pathogenesis is needed to further improve anti-cancer therapeutics as well as exosome-based cancer diagnostics.

## Figures and Tables

**Table 1:** Representative studies on the immunogenicity of tumor-derived exosomes and tumor exosome-based cancer vaccines.

Parental tumor type/exosome source	Exosome application/modification	Model	Results	References
Mouse mammary adenocarcinoma, melanoma, mesothelioma, mastocytoma, human melanoma	BMDC pulsed with exo were injected into mice with established tumor	Mouse	Exo transfer tumor antigen to DC, induce CD8+ T cell-dependent antitumor effects on both syngeneic and allogeneic mouse tumors	[33]

Malignant effusions of melanoma patients	MDDCs-pulsed exo were used to stimulate lymphocytes	Human *ex vivo *	DCs pulsed with exo cross-present mart-1 antigen to and expand antigen-specific CTLs	[32]

Human malignant glioma	Human DCs were incubated with exo	Human *ex vivo *	DCs incubated with exo activate glioma-specific CTL which kills autologous glioma cells *in vitro *	[36]

Human CEA+ colon, lung carcinoma	Exo were isolated from heat-stressed tumor cells	Mouse, Human *ex vivo *	Exo immunization efficiently prime antigen-specific CTL with antitumor effects in mice; exo-pulsed autologous DCs from CEA+ cancer patients induce antigen-specific CTL response	[34]

Mouse B lymphoma	Parental cells were heat-shocked	Mouse	Exo induce DC maturation and stimulate both protective and therapeutic antitumor immune responses	[40]

Mouse colon carcinoma and melanoma	Parental cells were heat-treated	Mouse	Exo contain elevated levels of Hsp70, elicit Th1 response and therapeutically regress established autologous and allogeneic tumors	[41]

Mouse melanoma	Parental cells were engineered to express membrane-bound Hsp 70	Mouse	Exo stimulate Th1 and CTL response more efficiently than exo derived from heat-shocked cells expressing cytoplasmic Hsp70	[42]

Mouse lung carcinoma	Parental cells were heat-stressed	Mouse	Exo contain enriched chemokines, attract/activate DCs and T cells more potently and induce antitumor response	[43]

Human CEA+ tumor cells	Parental cells were transfected with AdhIL-18	Human *ex vivo *	Exo/IL-18 chemoattract DCs and T cells and enhance Th1 cytokine release. Exo/IL18-pulsed DCs induced potent CTL response	[37]

Mouse OVA+ thymoma	Parental cells were transfected with AdmIL-12	Mouse	Vaccination of exo/IL-2 induces antigen-specific Th1 and CTL responses and inhibits tumor growth	[39]

Human renal cancer	Parental cells were modified to express GPI-IL-12	*In vitro*	Exo/IL-12 promote IFN-*γ* release and the induction of antigen-specific CTLs	[38]

Mouse lymphoma	Exo were surfaced anchored with the superantigen SEA by protein transfer	Mouse	Immunization with exo/SEA-TM efficiently inhibits tumor growth and induces tumor-specific CTLs	[44]

Mouse fibrosarcoma	OVA antigen was targeted to exo membrane by transfecting parental cells with OVA coupled to lactadherin C1C2 domain	Mouse	Tumors secreting exo-bound OVA elicit a stronger anti-OVA response and grow slowly *in vivo *	[45]

Human ovarian cancer ascites	Exo were purified from malignant ascites and quality assessed	Preceding of a clinical trial	A method for the preparation of GMP-grade exosomes used in combination of mature DCs for a clinical trial is described	[46]

Ascites from colorectal cancer patients	Exo were purified and used to immunize patients either alone or with GM-CSF	Phase I clinical trial	Exo therapy is well-tolerated; exo plus GM-CSF induce beneficial tumor-specific CTL responses in patients with colorectal cancer	[47]

Abbreviations: Exo, exosomes; MDDCs: monocyte-derived DCs; Ad: adenovirus; GM-CSF: granulocyte-macrophage colony-stimulating factor.
